# Resistance of *Vibrio cholera* to antibiotics that inhibit cell wall synthesis: A systematic review and meta-analysis

**DOI:** 10.3389/fphar.2023.1027277

**Published:** 2023-03-20

**Authors:** Hossein Nateghizad, Rojina Sajadi, Ali Shivaee, Omid Shirazi, Mohadeseh Sharifian, Danyal Abbasi Tadi, Kumarss Amini

**Affiliations:** ^1^ Department of Biology, Faculty of Basic Sciences, East of Tehran Branch, Islamic Azad University, Tehran, Iran; ^2^ Department of Microbiology, Shahid Beheshti University of Medical Sciences, Tehran, Iran; ^3^ Department of Veterinary medicine, Science and Research Branch, Islamic Azad University, Tehran, Iran; ^4^ Department Of Veterinary, Azad University Of Shahr-E Kord, Shahrekord, Iran; ^5^ Department of Microbiology, Saveh Branch, Islamic Azad University, Saveh, Iran

**Keywords:** antibiotic resistance, resistance, *Vibrio choler*a, *V. cholera*, meta-analysis

## Abstract

**Objective:** Cholera is a challenging ancient disease caused by *Vibrio cholera* (*V. cholera)*. Antibiotics that prevent cell wall synthesis are among the first known antibiotic groups. Due to its high consumption, *V. cholera* has developed resistance to the majority of antibiotics in this class. Resistance to recommended antibiotics for the treatment of *V. cholera* has also increased. In light of the decrease in consumption of certain antibiotics in this group that inhibit cell wall synthesis and the implementation of new antibiotics, it is necessary to determine the antibiotic resistance pattern of *V. cholera* and to employ the most effective treatment antibiotic.

**Method:** An comprehensive systematic search for relevant articles was conducted in PubMed, Web of Science, Scopus, and EMBASE through October 2020. Stata version 17.1 utilized the Metaprop package to execute a Freeman-Tukey double arcsine transformation in order to estimate weighted pooled proportions.

**Results:** A total of 131 articles were included in the meta-analysis. Ampicillin was the most investigated antibiotic. The prevalence of antibiotic resistance was in order aztreonam (0%), cefepime (0%), imipenem (0%), meropenem (3%), fosfomycin (4%), ceftazidime (5%), cephalothin (7%), augmentin (8%), cefalexin (8%), ceftriaxone (9%), cefuroxime (9%), cefotaxime (15%), cefixime (37%), amoxicillin (42%), penicillin (44%), ampicillin (48%), cefoxitin (50%), cefamandole (56%), polymyxin-B (77%), carbenicillin (95%) respectively.

**Discussion:** Aztreonam, cefepime, and imipenem are the most efficient *V. cholera* cell wall synthesis inhibitors. There has been an increase in resistance to antibiotics such as cephalothin, ceftriaxone, amoxicillin, and meropenem. Over the years, resistance to penicillin, ceftazidime, and cefotaxime, has decreased.

## Introduction

Koch isolated and described the Gram-negative *bacillus Vibrio* cholera (*V. cholera*) in the late nineteenth century (Dengo-Baloi et al., 2017). As a foodborne and waterborne pathogen, *V. cholera* can cause an acute intestinal infection as severe watery diarrhea in humans (Mukhopadhyay et al., 1998; Yuan et al., 2022). Virulence factors include toxin-related fimbria and cholera toxins (Ngandjio et al., 2009; Awuor et al., 2020a). In areas with poor sanitation and no clean water, *V. cholera* can be endemic, epidemic, or pandemic (Mukhopadhyay et al., 1998). This bacterium causes 2.9 million cholera cases and 95,000 deaths annually (Onohuean et al., 2022). According to the O antigen, *V. cholera* species are divided into 206 serotypes. Although the O1 and O139 serotypes are linked to epidemic cholera and non-agglutinating *V. cholera* (NOVC), which are negative to the O1 and O139 antigens, they cause infrequent severe illnesses (Liu et al., 2022; Onohuean et al., 2022). Seventh cholera pandemics linked to O1 and O139 *V. cholera* serotypes. The disease is a major issue in Asia, Africa, and Latin America (Abera et al., 2010; Alam et al., 2012). *V. cholera* infections can be mild, moderate, or severe in endemic areas (rapidly deadly diarrhea) (Araj et al., 1994; Anand et al., 1996).

Early treatment with an oral rehydration salts (ORS) solution, including glucose, potassium chloride, sodium chloride, and trisodium citrate, are critical for cholera patients with moderate watery diarrhea (Araj et al., 1994). Severe cholera dehydration requires intravenous rehydration and appropriate antibiotics to shorten the disease’s period (Araj et al., 1994; Baddam et al., 2020).

Although antibiotic susceptibility testing (AST) of *V. cholera* was not suggested in the past due to the low resistance of *V. cholera* to common antibiotics (Bag et al., 1998; Baddam et al., 2020), the development of resistance to tetracycline, a common antibiotic used in the treatment of *V. cholera* infection, is becoming increasingly widespread throughout the world (Bakhshi and Pourshafie, 2009). These resistant strains have been responsible for severe epidemics in Latin America, Tanzania, Bangladesh, and Zaire (Bag et al., 1998). However, various isolates of *V. cholera* resistant to antibiotics have been reported worldwide. Antibiotics that target the cell wall were once the first-line treatment for infections, but their use has decreased as bacterial resistance to these antibiotics has increased over time. Globally, the prevalence of *V. cholera* resistance to cell wall-active antibiotics has not been thoroughly studied. Replacing less effective antibiotics is necessary for more effective treatment, which has already increased the prevalence of resistance.

The tetracycline antibiotic class has long been the most effective for treating cholera. Non-etheless, earlier research revealed a global increase in *V. cholera* strains resistant to tetracycline (Dengo-Baloi et al., 2017). Additionally, studies have demonstrated that fluoroquinolone resistance in *V. cholera* strains began to increase in July 1996 (Mukhopadhyay et al., 1998; Yuan et al., 2022). A previously published study suggested erythromycin as a tetracycline alternative in small children and pregnant women. Furazolidone and nalidixic acid have traditionally been used as cholera treatments.

Nevertheless, due to the high level of resistance found in *V. cholera* isolates, these antibiotics are currently less effective (Ngandjio et al., 2009; Awuor et al., 2020b). Due to the lack of meta-analyses concerning antibiotics resistant to the *V. cholera* cell wall, we decided to explore newer antibiotics in terms of the prevalence of resistance as well as the global resistance pattern and the resistance trend over time in all *V. cholera* serotypes. This study’s outcomes can potentially improve the global antimicrobial resistance situation significantly.

## Methods

### Search strategy

The comprehensive systematic search of relevant articles through four electronic databases, including PubMed, Web of Science, Scopus, and EMBASE, with two researchers independently until October 2020. The search was performed using “*V. cholera*” and “Antibiotic resistance” related keywords. Obtained articles have been merged in EndNote X20 (Thomson Reuters, NY, United States), and duplicates were removed. The search syntax is available in Supplementary Material.

### Selection criteria and data extraction

The screening and selection of articles procedures were performed in Rayyan online software. Two authors (A. Sh and O. Sh) independently reviewed all records’ titles, abstracts, and full texts. They removed irrelevant articles, and the third author (M. Sh) solved disagreements. The exclusion criteria were as follows: review articles, case reports, congress abstracts, studies with ambiguous results, not the English language, sample size of fewer than three isolates, duplicate publications, and studies of antimicrobial resistance of other than *V. cholera* species.

The extracted information from each included study was: first author, year of publication, country, sample source (clinical or environmental isolates), serogroups, the total number of isolates (sample size), AST method, and the number of resistant isolates for each antibiotic.

### Quality assessment

The quality assessment of included studies was performed by two reviewers (R. Sa, and O. Sh) independently using an adapted version of the Newcastle-Ottawa assessment scale adapted for cross-sectional studies. Each study received a score ranging from 0 to 8 (5 points or higher: high quality, three or 4: medium quality, 2 points or lower: low quality).

### Statistical analysis

Due to the high number of zero prevalence in antibiotic resistance reports, the Freeman Tukey double arcsine conversion was conducted on the data using the metaprop command to estimate the weighted pooled proportion of resistance (WPR) in STATA software (version. 17.1), which range is from 0.00 to 1.00. A random-effects model was used for pooling effect size. As a measure of heterogeneity, the tau-squared and I^2^ were considered. The Egger regression test was used to determine the effect of small studies or publication bias. Subgroup analyses were conducted using the following variables to find the sources of variation: country, continent, country development status (World Economic Situation and Prospects, classification), publication year group (1970–2000, 2001–2010, 2011–2020), source of *V. cholera* isolation, AST method (Disc and Gradient methods), interpretation guideline (CLSI, Non-CLSI), and serogroups (O1/O139, Non-O1/O139).

## Results

The systematic search identified two thousand three hundred sixteen records, and 849 duplicates were removed. According to titles and abstracts screening, 1,141 articles were excluded, and according to the full-text evaluation, 195 articles were considered irrelevant. Finally, 131 articles published between 1980 and 2020 were included in this meta-analysis [Abera et al. (2010), Alam et al. (2012), Amita et al. (2003), Anand et al. (1996), Araj et al. (1994), Baddam et al. (2020), Bag et al. (1998), Bakhshi et al. (2014), Bakhshi and Pourshafie (2009), Bakhshi et al. (2008), Balaji et al. (2013), Ballal and Shivananda (2002), Bani et al. (2007); Barati et al. (2015), Basu et al. (2000), Bhat et al. (2012), Bhattacharya et al. (2015), Bhattacharya et al. (2012), Bhotra et al. (2017), Bhowmick et al. (2007), Bier et al. (2015), Borkakoty et al. (2012), Campos et al. (2004), Ceccarelli et al. (2016); Ceccarelli et al. (2011), Chakraborty et al. (2001), Chandrasekhar et al. (2008), Chatterjee et al. (2007), Chhotray et al. (2002), Chomvarin et al. (2013), Chomvarin et al. (2012), Chowdhury et al. (2016), Colombo et al. (1993), Dalsgaard et al. (1998), Dalsgaard et al. (2000), Dalsgaard et al. (1996), Das et al. (2016), Das et al. (2011), Das et al. (2008), Dengo-Baloi et al., 2017, Dey et al. (2014), Dua et al. (2018), Echeverria et al. (1983), Eibach et al. (2016); Faruque et al. (2000); Fazil et al. (2011); Feglo and Sewurah (2018), Fernandez-Abreu et al. (2017), Folgosa et al. (2000), Garg et al. (2000), Goel et al. (2010), Gupta et al. (2016), Ibarra and Alvarado (2007), Iramiot et al. (2019); Islam et al. (2011); Ismail et al. (2013); Jain et al. (2016); Jaiswal et al. (2015), Kacou-N’douba et al. (2012), Kaistha et al. (2005), Kar et al. (2015), Karki et al. (2010), Kingston et al. (2009), Koley et al. (2014), Kondo et al. (2001), Krishna et al. (2006), Kumar et al. (2014), Kutar et al. (2013), Luo et al. (2013), Mandal et al. (2010), Mandal et al. (2009), Mandomando et al. (2007), Marashi et al. (2012), Mohapatra et al. (2007), Sarkar et al. (2019), Moyo et al. (2011), Mukhopadhyay et al. (1995), Okuda et al. (2007), Oyofo et al. (2002b), Oyofo et al. (2002a), Pal et al. (2006), Pal et al. (2017), Pan et al. (2008), Parveen et al. (2003), Patrick et al. (2012), Rahbar et al. (2010), Ranjbar et al. (2016), Rashed et al. (2012), Rezaie et al. (2017), Roy et al. (2012), Saidi et al. (2014), Sambe-Ba et al. (2017), Sedaghat et al. (2012), Shakya et al. (2012), Sharma et al. (2007), Talkington et al. (2011), Torane et al. (2016), Tran et al. (2012), Vijayalakshmi et al. (1997), Vinothkumar et al. (2018), Akoachere et al. (2013), Akoachere and Mbuntcha (2014), Alaoui et al. (2010), Al-Hilu et al. (2019), Amau et al. (1988), Baron et al. (2017), Baron et al. (2016), Bhanumathi et al. (2003), Bidinost et al. (2004), Bier et al. (2015), Campos et al. (2004), Ceccarelli et al. (2016), Chakraborty et al. (2001), Chomvarin et al. (2013), Dalsgaard et al. (1996), Eja et al. (2006), Falcao et al. (1998), Ibarra and Alvarado (2007), Imziln and Hassani (1994), Isaac-Márquez et al. (1998), Jagadeeshan et al. (2009), Kiiru et al. (2013), Kumar and Lalitha (2013), Li et al. (2011), Mandal et al. (2010), Mercy et al. (2014), Mishra et al. (2011), Mohapatra et al. (2007), Odjadjare and Igbinosa (2017), Onyuka et al. (2011), Osawa et al. (2020), Ottaviani et al. (2018), Prabhu et al. (2007), Radu et al. (2002), Kutar et al. (2013), Singh et al. (2018), Song et al. (2013), Wang et al. (2016), Waturangi et al. (2013), Zachariah et al. (2002), Kumaran and Citarasu (2016)].

**FIGURE 1:**
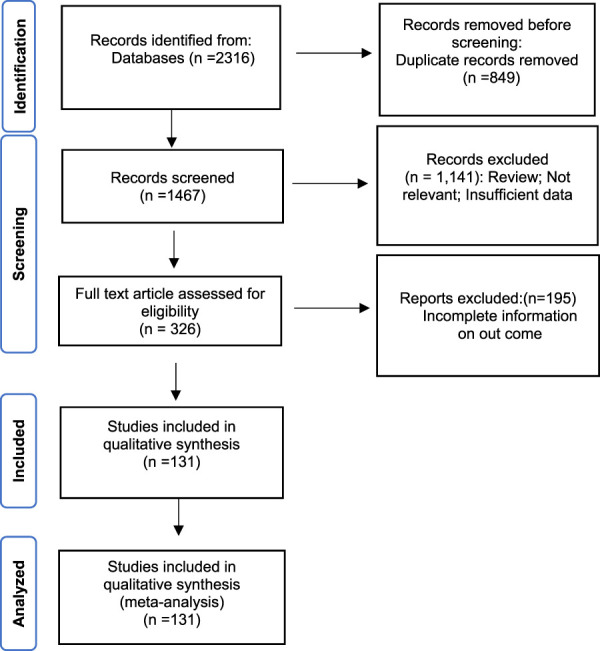
The study Prisma flow diagram.

**FIGURE 2:**
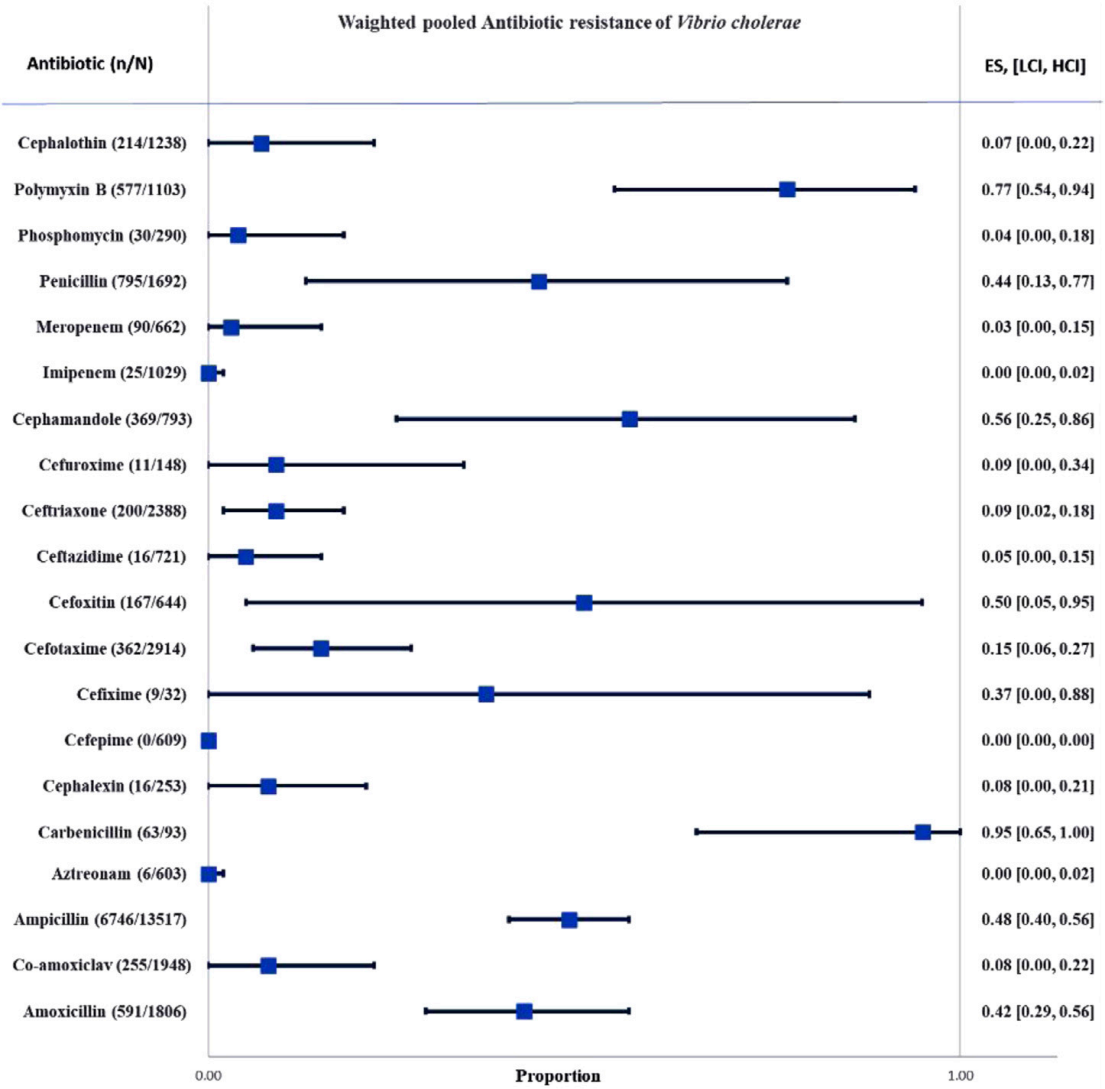
Forest plot of the weighted pooled proportion of antimicrobial-resistant *Vibrio cholera*. Abbreviations; Effect size or proportion of resistance (ES), number of resistant isolates (n), number of isolates (N), high confidence interval (HCI), low confidence interval (LCI).

The whole search and selection prudcidure are summarized in prizma flow diagram (Figure 1). The included studies have been conducted in 40 countries. Descriptive statistics of included articles are summarized in Table 1. The WPR of *V. cholera* to each antibiotic and the result of the egger test are summarized in Table 2. The total number of *V. cholera* isolates, the number of resistant isolates, and the WPR of each antibiotic are mentioned in the forest plot (Figure 2). The *V. cholera* WPR to the antibiotics in each continent is summarized in Table 3. The evolution of antibiotic resistance over time is depicted in Table 4. Also, the WPR in developed and developing countries is summarized in Table 5. The search syntax and all extracted data from included articles are available in Supplementary Material.

**TABLE 1:** Descriptive statistics of included articles.

	Categories	Number of articles	Number of isolates
Continents	Africa	33	3,727
	Asia	154	45,559
	Europe	10	1,018
	Multi Continent	5	200
	North America	8	498
	South America	7	216
Year groups	1970–2000	33	5,969
	2001–2010	75	26,538
	2011–2020	109	18,711
AST methods	Disc	183	45,620
	Gradient	34	5,598
AST guidelines	CLSI	165	26,048
	Non-CLSI	52	25,170
Classification based on the development state of countries	Developed	16	1,561
	Developing	192	48,505
	N. A	9	1,152

**TABLE 2:** Proportion of *Vibrio cholera* antibiotic-resistant isolates.

Antibiotics	Proportion [LCI, HCI]	Heterogeneity (I^2^)	*p*-value	Egger
Amoxicillin	0.42 [0.29, 0.56]	96.95	0.00	0.05
Agumentin	0.08 [0.00, 0.22]	98.11	0.00	0.86
Ampicillin	0.48 [0.40, 0.56]	98.77	0.00	0.30
Aztreonam	0.00 [0.00, 0.02]	49.25	0.08	0.36
Carbenicillin	0.95 [0.65, 1.00]	85.02	0.00	0.01
Cefalexin	0.08 [0.00, 0.21]	86.27	0.00	0.15
Cephalothin	0.07 [0.00, 0.22]	97.81	0.00	0.22
Cefepime	0.00 [0.00, 0.00]	0.00	0.90	0.35
Cefixime	0.37 [0.00, 0.88]	86.80	0.00	0.20
Cefotaxime	0.15 [0.06, 0.27]	97.94	0.00	0.09
Cefoxitin	0.50 [0.05, 0.95]	99.30	0.00	0.21
Ceftazidime	0.05 [0.00, 0.15]	90.63	0.00	0.07
Ceftriaxone	0.09 [0.02, 0.18]	96.90	0.00	0.15
Cefuroxime	0.09 [0.00, 0.34]	89.81	0.00	0.22
Cefamandole	0.56 [0.25, 0.86]	98.64	0.00	0.45
Imipenem	0.00 [0.00, 0.02]	79.95	0.00	0.53
Meropenem	0.03 [0.00, 0.15]	94.48	0.00	0.11
Penicillin	0.44 [0.13, 0.77]	99.40	0.00	0.53
Fosfomycin	0.04 [0.00, 0.18]	93.03	0.00	0.32
Polymyxin B	0.77 [0.54, 0.94]	98.24	0.00	0.20

95% Confidence Intervals were considered, and *p*-value ≤ 0.05 was considered statistically significant. Abbreviations; HCI, High confidence interval; LCI, Low confidence interval; I^2,^, I-squared.

**TABLE 3:** The Proportion of *V. cholera* antibiotic-resistant isolates among continents.

	Africa	Asia	Europe	Multi-continent	North America	South America	Heterogeneity between groups
Amoxicillin	(197/835) 0.43 [0.22, 0.67]	(301/761) 0.50 [0.30, 0.69]	(89/146) 0.61 [0.53, 0.69]	(4/64) 0.06 [0.01, 0.14]	NA	NA	*p <* 0.001
Augmentin	(60/1,158) 0.08 [0.00, 0.24]	(192/450) 0.17 [0.00, 0.56]	(3/290) 0.00 [0.00, 0.04]	NA	(0/50) 0.00 [0.00, 0.03]	NA	*p =* 0.096
Ampicillin	(859/2,366) 0.60 [0.36, 0.81]	(5,209/9,480) 0.49 [0.40, 0.59]	(135/525) 0.17 [0.03, 0.37]	(123/200) 0.54 [0.02, 1.00]	(303/730) 0.41 [0.20, 0.63]	(117/216) 0.43 [0.14, 0.74]	*p* = 0.088
Aztreonam	NA	(6/591) 0.00 [0.00, 0.03]	(0/12) 0.00 [0.00, 0.14]	NA	NA	NA	*p* = 0.868
Carbenicillin	NA	(28/28) 1.00, (95% CI, [0.93, 1.00]	NA	NA	(35/65) 0.54 [0.42, 0.66]	NA	*p <* 0.001
Cephalexin	(0/16) 0.00 [0.00, 0.10]	(16/237) 0.09 [0.00, 0.25]	NA	NA	NA	NA	*p* = 0.161
Cefepime	NA	(0/460) 0.00 [0.00, 0.00]	(0/149) 0.00 [0.00, 0.01]	NA	NA	NA	*p* = 0.667
Cefixime	(4/6) 0.67 [0.24, 0.99]	(5/26) 0.28 [0.00, 0.90]	NA	NA	NA	NA	*p* = 0.338
Cefotaxime	(124/483) 0.22 [0.00, 0.79]	(238/2026) 0.23 [0.10, 0.39]	(0/290) 0.00 [0.00, 0.00]	NA	(0/115) 0.00 [0.00, 0.02]	NA	*p <* 0.001
Cefoxitin	(125/125) 1.00 [0.99, 1.00]	(42/511) 0.29 [0.03, 0.66]	NA	NA	NA	(0/8) 0.00 [0.00, 0.20]	*p <* 0.001
Ceftazidime	NA	(16/572) 0.10 [0.00, 0.28]	(0/149) 0.00 [0.00, 0.01]	NA	NA	NA	*p =* 0.004
Ceftriaxone	(41/825) 0.16 [0.00, 0.56]	(86/1,171) 0.07 [0.00, 0.21]	(0/77) 0.00 [0.00, 0.02]	(61/102) 0.60 [0.50, 0.69]	(0/65) 0.00 [0.00, 0.03]	(12/148) 0.07 [0.01, 0.15]	*p <* 0.001
Cefuroxime	(7/137) 0.03 [0.00, 0.17]	(4/11) 0.30 [0.05, 0.63]	NA	NA	NA	NA	*p =* 0.025
Cephamandole	(191/442) 0.79 [0.17, 1.00]	(167/203) 0.81 [0.43, 1.00]	NA	NA	NA	(11/148) 0.06 [0.02, 0.12]	*p <* 0.001
Imipenem	NA	(21/705) 0.00 [0.00, 0.05]	(4/248) 0.01 [0.00, 0.04]	NA	(0/76) 0.00 [0.00, 0.02]	NA	*p =* 0.749
Meropenem	NA	(0/32) 0.00 [0.00, 0.05]	(2/191) 0.00 [0.00, 0.03]	NA	(88/439) 0.20 [0.16, 0.24]	NA	*p <* 0.001
Penicillin	(57/58) 0.98 [0.93, 1.00]	(730/1,626) 0.23 [0.01, 0.60]	NA	(8/8) 1.00 [0.80, 1.00]	NA	NA	*p <* 0.001
Fosfomycin	NA	(0/80) 0.00 [0.00, 0.02]	(29/146) 0.20 [0.14, 0.27]	(1/64) 0.01 [0.00, 0.05]	NA	NA	*p <* 0.001
Polymyxin B	(57/336) 0.16 [0.12, 0.20]	(436/677) 0.74 [0.50, 0.93]	NA	(58/64) 0.92 [0.83, 0.98]	(26/26) 1.00 [0.93, 1.00]	NA	*p <* 0.001
Cephalothin	(190/682) 0.17 [0.14, 0.20]	(10/266) 0.10 [0.00, 0.38]	NA	(1/64) 0.01 [0.00, 0.06]	(2/65) 0.03 [0.00, 0.09]	(11/161) 0.04 [0.00, 0.16]	*p <* 0.001

Legend: The heatmap of weighted proportions of resistance 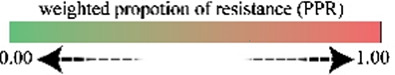, NA, Not Available: (n/N) proportion (95% Confidence interval, [Low confidence interval, High Confidence interval].

**TABLE 4:** Pooled Proportion of *Vibrio cholera* antibiotic resistance over the years.

	1970–2000	2001–2010	2011–2020	Heterogeneity between groups
Amoxicillin	(121/575), 0.18 [0.00, 0.52]	(363/998), 0.51 [0.33, 0.69]	(107/233), 0.50 [0.30, 0.70]	*p =* 0.207
Augmentin	(0/19), 0.00 [0.00, 0.09]	(47/358), 0.12 [0.08, 0.16]	(208/1,571), 0.09 [0.00, 0.27]	*p* = 0.078
Ampicillin	(1,123/2054), 0.44 [0.25, 0.65]	(3,242/5,005), 0.50 [0.37, 0.64]	(2,381/6,458), 0.48 [0.37, 0.59]	*p* = 0.891
Aztreonam	(0/19), 0.00 [0.00, 0.09]	(0/12), 0.00 [0.00, 0.14]	(6/572), 0.01 [0.00, 0.03]	*p* = 0.988
Carbenicillin	(52/82), 0.89 [0.44, 1.00]	NA	(11/11), 1.00 [0.83, 1.00]	*p* = 0.509
Cephalexin	(4/22), 0.18 [0.04, 0.37]	(3/60), 0.03 [0.00, 0.11]	(9/171), 0.09 [0.00, 0.33]	*p* = 0.161
Cefepime	NA	NA	(0/609), 0.00 [0.00, 0.00]	NA
Cefixime	NA	NA	(9/32), 0.37 [0.00, 0.88]	NA
Cefotaxime	(32/188), 0.23 [0.00, 0.65]	(83/1,133), 0.26 [0.08, 0.50]	(247/1,593), 0.10 [0.01, 0.26]	*p* = 0.401
Cefoxitin	(17/27), 0.62 [0.42, 0.80]	(20/32), 0.62 [0.45, 0.79]	(130/585), 0.51 [0.00, 1.00]	*p =* 0.957
Ceftazidime	(12/36), 0.41 [0.00, 1.00]	(0/44), 0.00 [0.00, 0.04]	(4/641), 0.00 [0.00, 0.03]	*p* = 0.294
Ceftriaxone	(0/65), 0.00 [0.00, 0.03]	(81/598), 0.08 [0.00, 0.24]	(119/1725), 0.11 [0.01, 0.25]	*p* = 0.008
Cefuroxime	NA	NA	(11/148), 0.09 [0.00, 0.34]	NA
Cefamandole	NA	(103/497), 0.17 [0.05, 0.35]	(266/296), 0.92 [0.71, 1.00]	*p <* 0.001
Imipenem	(0/19), 0.00 [0.00, 0.09]	NA	(25/1,010), 0.00 [0.00, 0.02]	*p* = 0.916
Meropenem	NA	(0/32), 0.00 [0.00, 0.05]	(90/630), 0.04 [0.00, 0.19]	*p* = 0350
Penicillin	(57/58), 0.98 [0.93, 1.00]	(614/730), 0.84 [0.81, 0.87]	(124/904), 0.19 [0.09, 0.30]	*p* < 0.001
Fosfomycin	(30/210), 0.06 [0.00, 0.25]	NA	(0/80), 0.00 [0.00, 0.02]	*p* = 0.138
Polymyxin B	(77/83), 0.94 [0.83, 1.00]	(191/563), 0.62 [0.22, 0.95]	(309/457), 0.81 [0.52, 0.99]	*p* = 0.160
Cephalothin	(3/513), 0.00 [0.00, 0.03]	(203/698), 0.07 [0.00, 0.36]	(8/27), 0.33 [0.00, 0.99]	*p* = 0.214

Legend: The heatmap of weighted pooled proportions of resistance 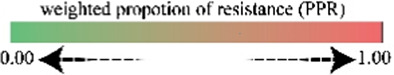, NA: not available; (n/N), proportion (95% Confidence interval, [Low confidence interval, High Confidence interval].

**TABLE 5:** Pooled proportion of *Vibrio cholera* antibiotic resistance in developing and developed countries.

	Developing countries	Developed countries	Heterogeneity between groups
Amoxicillin	(498/1,596) 0.47 [0.32, 0.62]	(89/146) 0.61 [0.53, 0.69]	*p <* 0.001
Augmentin	(251/933) 0.14 [0.01, 0.35]	(4/299) 0.00 [0.00, 0.05]	*p =* 0.001
Ampicillin	(5,919/10,799) 0.52 [0.43, 0.60]	(677/1709) 0.24 [0.08, 0.44]	*p =* 0.041
Aztreonam	(3/573) 0.00 [0.00, 0.01]	(3/30) 0.07 [0.00, 0.21]	*p =* 0.011
Carbenicillin	(63/93) 0.95 [0.65, 1.00]	NA	NA
Cephalexin	(16/244) 0.09 [0.00, 0.24]	(0/9) 0.00 [0.00, 0.18]	*p =* 0.335
Cefepime	(0/460) 0.00 [0.00, 0.00]	(0/149) 0.00 [0.00, 0.01]	*p =* 0.667
Cefixime	(9/32) 0.37 [0.00, 0.88]	NA	NA
Cefotaxime	(362/2,624) 0.19 [0.08, 0.33]	(0/290) 0.00 [0.00, 0.00]	*p <* 0.001
Cefoxitin	(167/644) 0.50 [0.05, 0.95]	NA	NA
Ceftazidime	(16/572) 0.09 [0.00, 0.25]	(0/149) 0.00 [0.00, 0.01]	*p =* 0.004
Ceftriaxone	(133/1,493) 0.08 [0.01, 0.19]	(0/77) 0.00 [0.00, 0.02]	*p =* 0.008
Cefuroxime	(11/148) 0.09 [0.00, 0.34]	NA	NA
Cefamandole	(369/793) 0.56 [0.25, 0.86]	NA	NA
Imipenem	(21/781) 0.00 [0.00, 0.03]	(4/248) 0.01 [0.00, 0.04]	*p <* 0.001
Meropenem	(88/471) 0.18 [0.14, 0.21]	(2/191) 0.00 [0.00, 0.03]	*p =* 0.913
Penicillin	(749/1,377) 0.40 [0.07, 0.79]	(38/307) 0.12 [0.09, 0.16]	*p <* 0.001
Fosfomycin	(0/80) 0.00 [0.00, 0.02]	(29/146) 0.20 [0.14, 0.27]	*p <* 0.001
Polymyxin B	(519/1,039) 0.75 [0.51, 0.94]	NA	NA
Cephalothin	(213/1,174) 0.08 [0.00, 0.27]	NA	NA

Legend: The heatmap of weighted pooled proportions of resistance 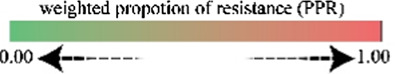, NA: not available; (n/N) Proportion (95% Confidence interval, [Low confidence interval, High Confidence interval].

### Resistance to penicillin’s

The *V. cholera* WPR to penicillin is 0.44 (95% CI, (95% CI, [0.13, 0.77]), and the heterogeneity was significant (I^2^ = 99.40, *p* < 0.01). The highest WPR among countries was observed in Bangladesh (WPR; 0.14). In most studies, AST was performed by the disc diffusion method. WPR in CLSI and non-CLSI method subgroups was 0.36 and 0.84, respectively. The serogroup heterogeneity and isolate sources subgroups were insignificant (*p* > 0.05).

*V. cholera* WPR to ampicillin is 0.48 (95% CI [0.40, 0.56]), and heterogeneity was significant (I^2^ = 98.77, *p* < 0.01). The countries with the highest WPR were France, Nepal, and Iran (WPR; 1.00, 1.00, 0.97, respectively). Heterogeneity between the source of isolates, serogroups, and AST method subgroups was insignificant (*p* > 0.05).

The *V. cholera* WPR to amoxicillin is 0.42 (95% CI [0.29, 0.56]) and heterogeneity was significant (I^2^ = 96.95, *p* < 0.01). Cameroon and India have the highest WPR among countries (WPR; 0.52, 0.43, respectively). The resistance rate of clinical isolates was much higher than environmental isolates (WPR; 0.43, 0.35, respectively). The heterogeneity of subgrouping based on the AST method, AST guidelines, and serogroups were insignificant (*p* > 0.05).

The *V. cholera* WPR to carbenicillin is 0.95 (95% CI [0.65, 1.00]), and heterogeneity was significant (I^2^ = 85.02, *p* < 0.01). The highest WPR was in Malaysia and India (WPR; 1.00, 1.00, respectively). The WPR in clinical isolates was much higher than in environmental isolates (WPR; 1.00, 0.54, respectively). The WPR of the O1/O139 serogroup was higher than the non-O1/O139 serogroup (WPR; 1.00, 0.54, respectively). All the studies have used CLSI guidelines. The heterogeneity of subgrouping based on the AST method was insignificant (*p* > 0.05).

The *V. cholera* WPR to augmentin (amoxicillin/clavulanate) is 0.08 (95% CI [0.00, 0.22]), and the heterogeneity was significant (I^2^ = 98.11, *p* < 0.01). India has the highest resistance rate (WPR). The heterogeneity of subgrouping based on the isolate’s sources, serogroups, and AST guidelines was insignificant (*p* > 0.05).

### Cephalosporins (1st gen)

#### Resistance to cefalexin

The *V. cholera* WPR to cefalexin is 0.08 (95% CI [0.0.0, 0.21]), and heterogeneity between reports was significant (I^2^ = 86.27, *p* < 0.01). Every included sample was a clinical isolate. The heterogeneity of subgrouping based on the countries, AST methods, AST guidelines, and serogroups was insignificant (*p* > 0.05). The *V. cholera* WPR to cephalothin is 0.07 (95% CI [0.00–0.22]), and heterogeneity was significant (I^2^ = 97.81, *p* < 0.01). India, Brazil, and Morocco have the highest WPR among countries (WPR; 0.62, 0.06, and 0.17, respectively). Most of the articles have used the disk diffusion method for AST. Heterogeneity between the isolated sources was insignificant (*p* = 0.969).

### Cephalosporins (2nd gen)

The *V. cholera* WPR to cefoxitin is 0.50 (95% CI [0.05–0.95]), and heterogeneity was significant (I^2^ = 99.30, *p* < 0.01). The highest WPR was in Bangladesh (WPR; 0.01). The *V. cholera* WPR to cefuroxime was 0.09 (95% CI [0.00 to 0.34]), and the heterogeneity was significant (I^2^ = 89.81, *p* < 0.01). The countries with the highest WPR were India and Ghana (WPR; 0.30, 0.17, respectively). The WPR of clinical isolates was higher than environmental isolates (WPR; 0.16, 0.00, respectively). Heterogeneity of subgrouping based on the AST method; the AST guidelines were insignificant (*p* > 0.05). All studies have used CLSI, and all isolates were O1/O139 serogroup.

The WPR of *V. cholera* to carbenicillin was 0.95 (95% CI [0.65, 1.00]), and the heterogeneity was significant (I^2^ = 85.02, *p* < 0.01). Malaysia and India have the highest WPRs (WPR; 1.00, 1.00, respectively). All of the studies have been in developed countries. Clinical isolates had a substantially higher WPR than environmental isolates (WPR; 1.00, 0.54, respectively). The WPR in O1/O139 serogroup was higher than the non-O1/O139 serogroup (WPR; 1.00 and 0.54, respectively). All studies have used CLSI as an AST guideline. The heterogeneity of subgrouping based on the AST methods was insignificant (*p* = 0.52).

### Cephalosporins (3rd gen)

The WPR of *V. cholera* to cefixime is 0.37 (95% CI [0.00–0.88]), and the heterogeneity was significant (I^2^ = 86.80, *p* < 0.01). Nigeria had the highest WPRs among countries (WPR; 0.67).

The heterogeneity between countries and sources of isolates was significant (*p* < 0.05). The heterogeneity between the AST method and serogroups was insignificant (*p* = 0.072). All studies used CLSI guidelines.

The WPR of *V. cholera* to cefotaxime is 0.15 (95% CI [0.06, 0.27]), and the heterogeneity was significant (I^2^ = 97.94, *p* < 0.01). The countries with the highest WPR were India, Bangladesh, and Germany (WPR; 0.21, 0.12, and 0.00, respectively). Heterogeneity of subgrouping based on the. the isolate sources, AST method, AST guidelines, and serogroups were insignificant (*p* > 0.05). The *V. cholera* WPR to ceftazidime is 0.05 (95% CI [0.00–0.15]), and the heterogeneity was significant (I^2^ = 90.63, *p* < 0.01). Malaysia and India have the highest WPR (WPR; 0.80, 0.12, respectively). The WPR in clinical isolates was higher than in environmental isolates (WPR; 0.02, 0.00, respectively). Most studies have investigated AST by disk diffusion method (WPR; 0.10). All studies have used CLSI as an AST guideline. The WPR of the O1/O139 serogroup was higher than the non-O1/O139 serogroup (WPR; 0.10 and 0.00, respectively). The *V. cholera* WPR to ceftriaxone is 0.09 (95% CI [0.02–0.18]), and the heterogeneity was significant (I^2^ = 96.90, *p* < 0.01). The countries with the highest WPR were Iran, Brazil, and India (WPR; 1.00, 0.07, 0.04, respectively). The WPR of clinical isolates was much more than environmental isolates (WPR; 0.14, 0.03, respectively). The heterogeneity of subgrouping based on the AST guidelines and serotypes was insignificant (*p* > 0.05).

### Cephalosporins (4th gen)

The *V. cholera* WPR to cefepime is 0.00 (95% CI [0.00, 0.00]), and the heterogeneity was insignificant (I^2^ = 0.00, *p* = 0.90).

### Resistance to carbapenems

The *V. cholera* WPR to imipenem is 0.00 (95% CI [0.00–0.02]), and heterogeneity was significant (I^2^ = 79.95, *p* < 0.01). India and Germany had the highest WPR (0.05 and 0.02, respectively). CLSI was utilized as a guideline in all studies. The heterogeneity of subgrouping based on the continents, AST method, AST guideline, isolated sources, and serogroups were insignificant (*p* > 0.05).

The *V. cholera* WPR to meropenem is 0.03 (95% CI [0.00, 0.15], and the heterogeneity was significant (I^2^ = 94.48, *p* < 0.01). Germany had the lowest WPR among countries (WPR; 0.00). The WPR in O1/O139 serogroup was higher than non-O1/O139 serogroup (WPR; 0.18 0.00 respectively).

### Resistance to aztreonam

The WPR of *V. cholera* to aztreonam is 0.00 (95% CI [0.00, 0.02]). The heterogeneity was insignificant (I^2^ = 49.25, *p* = 0.08).

### Resistance to fosfomycin

The *V. cholera* WPR to fosfomycin is 0.04 (95% CI [0.00–0.18]), and the heterogeneity was significant (I^2^ = 93.03, *p* < 0.01). Spain had the highest WPR among countries (WPR; 0.20), and Iran reported the lowest resistance rate. (WPR; 0.00). The WPR of clinical isolates was higher than environmental isolates (WPR; 0.00, 0.17, respectively). The WPR of the non-CLSI group was higher than the CLSI group (WPR; 0.00 and 0.20, respectively).

### Resistance to polymyxin B

The *V. cholera* WPR to polymyxin B is 0.77 (95% CI [0.54, 0.94]), and the heterogeneity was significant (I^2^ = 98.24, *p* < 0.01). The countries with the highest WPR were India and Iran (WPR; 0.86, 0.65, respectively). The WPR of the CLSI subgroup was higher than the non-CLSI group (WPR; 0.78, 0.16, respectively). The heterogeneity of subgrouping based on the serogroups and isolate sources was insignificant (*p* > 0.05).

## Discussion

Cholera is a historical, unresolved problem and a severe health-threatening infection, particularly in developing countries. This disease has been endemic throughout South Asia, notably in Bangladesh and India’s Ganges delta region (18). This condition expanded outside the Indian subcontinent *via* trade channels, resulting in worldwide pandemics with significant fatality rates (millions of deaths) (19). Antibiotics alone are ineffective against severe cholera. However, fluid replacement and antibiotic treatment are combined because antibiotics can reduce cholera bacteria in the feces and shorten the duration of the disease (19).

Due to *V. cholera*’s low resistance, the AST of these microorganisms was avoided in the past. (14, 15). However, the emergence of *β*-lactam-resistant species has had severe repercussions on managing infectious diseases globally (16). Tetracyclines have long been the antibiotic for treating severe cholera (15). Conversely, tetracycline-resistant *V. cholera* strains are spreading internationally (17). Severe epidemics in Latin America, Tanzania, Bangladesh, and Zaire have been linked to tetracycline-resistant strains (15). A meta-analysis with 52 studies has estimated the tetracycline and doxycycline resistance rate of *V. cholera* serotype O1 over 50% and 28%, respectively (20). This escalating resistance rate underscores the importance of regulating antibiotic prescriptions and discovering effective alternatives.

The current study investigated *V. cholera* cell wall-targeting antibiotic resistance patterns. This study revealed that carbenicillin (95%), and polymyxin B (77%) have the highest resistance rates. The ampicillin, penicillin, and amoxicillin resistance rates were 48%, 44%, and 42%, respectively. Adding clavulanate acid to amoxicillin reduced the proportion of resistant bacteria to 8%, which was more effective than amoxicillin alone.

The rates of resistance to the first generation of cephalosporins, such as cefalexin and cephalothin, were 8% and 7%, respectively. The percentages of resistance to the second generation of cephalosporins, including cefamandole, cefoxitin, and cefuroxime, were 50%, 56%, and 9%, respectively. Compared to the first generation of cephalosporins, *V. cholera* was more resistant to the second generation. Cefixime (37%), cefotaxime (15%), ceftazidime (5%), and ceftriaxone (9%) As members of the third generation of cephalosporins are more effective than second-generation cephalosporins. Cefepime is the only member of the fourth generation of cephalosporins studied in four articles evaluating 609 *V. cholera* isolates, and none of them were resistant to it.

The rate of aztreonam resistance was 0%, while the rate of fosfomycin resistance was 4%. Cefepime, aztreonam, and imipenem were the most efficient antibiotics against *V. cholera*. Non-etheless, the heterogeneity between reported imipenem resistance rates was substantial, and it appears necessary to perform AST before prescribing antibiotics to ensure the isolate’s susceptibility.

Considering sulfonamides are commonly used to treat HIV, TB, malaria, pneumonia, and febrile illness, Onohuean et al. (2022) estimated the prevalence of quinolone, tetracycline, and sulfonamide resistance genes to be 32.97 percent (95% CI [0.18–0.55]). This percentage is lower than that of polymyxin and higher than that of cephalosporins. An additional study was analyzed from a total of 139 articles involving 24,062 isolates of *V. cholera* O1/O139. Asia was the location of origin for 102 out of the total research. The WPR was calculated as follows: azithromycin had a 1%, erythromycin 36%, ciprofloxacin 3%, cotrimoxazole 79%, doxycycline 7%, and tetracycline had a success rate of 20% WPR. Between the years 1980 and 2020, there was a growth in drug resistance to cotrimoxazole, ciprofloxacin, and tetracycline (Liu et al., 2022).

According to the findings of Xin-hui Yuan’s research, there has been an increase in drug resistance in recent years, particularly to nalidixic acid, cotrimoxazole, furazolidone, and tetracycline. Between 2000 and 2020, however, resistance to antibiotics such as amoxicillin, ciprofloxacin, erythromycin, chloramphenicol, ampicillin, streptomycin, and ceftriaxone decreased. The frequency of doxycycline and ciprofloxacin resistance in *V. cholera* O1/O139 isolates significantly reduced from 2011 to 2020 compared to the frequency of these resistances from 2001 to 2010 (*p* < 0.05) (Yuan et al., 2022).

Based on subgroup analysis of continents, Africa and Asia had the highest proportion of resistant individuals. Patterns of resistance vary between developing and developed nations, and the resistance rate in developing nations was significantly higher than in developed nations. Even though developed countries had more resistant bacteria to amoxicillin, aztreonam, imipenem, and fosfomycin, developing countries had more resistant bacteria to augmentin, penicillin, ampicillin, cefotaxime, ceftazidime, and ceftriaxone.

Antibiotics to which *V. cholera* is highly resistant in developed nations have not been studied in developing countries. Cefixime, cefoxitin, cefuroxime, cefamandole, polymyxin B, cephalothin, and carbenicillin appear to have been taken off the list of *V. cholera* treatments. There has been an increase in resistance to antibiotics such as cephalothin, ceftriaxone, amoxicillin, and meropenem. Over the years, resistance to penicillin, ceftazidime, and cefotaxime has decreased. Because cholera was historically treated with numerous antibiotics, resistance to these antibiotics grew over time. For instance, penicillin resistance reached 98% during a specific time frame. After this increase in *V. cholera* infection treatment, it appears that this antibiotic was no longer considered. In recent years, the resistance to this antibiotic has decreased significantly, reaching 19% due to the lack of prescribing and use.

## Conclusion

Our findings indicate the importance of prescribing antibiotics accurately to control and prevent *V. cholera* antibiotic resistance. Following the disappointing emergence of antibiotic resistance to certain antibiotics, such as penicillin, these antibiotics were utilized less frequently, which resulted in a decrease in antibiotic resistance to these antibiotics in general. This event increases optimism that using old antibiotics will be effective if antibiotic use is controlled. In that order, the antibiotics with the least resistance to *V. cholera* were cefepime, aztreonam, imipenem, and meropenem. Due to the vastly different patterns of antibiotic resistance of *V. cholera* to these antibiotics in various geographic locations, it appears necessary to investigate the antibiotic resistance of the isolates.
