# Supplementary material for: Resistance of Vibrio cholera to antibiotics that inhibit cell wall synthesis: A systematic review and meta-analysis
Source: Front Pharmacol. 2023 Mar 20;14:1027277. doi: 10.3389/fphar.2023.1027277 (PMC10069679; doi:10.3389/fphar.2023.1027277)
Supplement: Supplementary file 1 [file Table1.docx]

**Search syntax:**

("Vibrio" OR "cholera" OR "Vibrio cholera*” OR "V. cholera*" OR " Vibriosis ") AND ("resistance" OR "antimicrobial resistance" OR "Antimicrobial-Drug Resistance" OR "drug resistance" OR "antibiotic resistance" OR "beta-lactams" OR " amoxicillin clavulanate” OR "fosfomycin" OR Amoxicillin" OR "Augmentin" OR "Ampicillin" OR "Aztreonam" OR "Bacitracin" OR "Cefotaxime" OR "Cefoxitin" OR "Ceftazidime" OR "Ceftriaxone " OR "Cefuroxime" OR "Cephamandole" OR "Imipenem" OR "Meropenem" OR "Penicillin" OR "Phosphomycin" OR "Polymyxin B" OR "Vancomycin" OR "Cephalothin) in the Title/Abstract/Keywords fields.
